# Neutrophils loaded NAD^+^ impede TLR4/NF-κB/NLRP3 pathway for sepsis treatment

**DOI:** 10.1016/j.mtbio.2025.102168

**Published:** 2025-08-04

**Authors:** Yingchun Zhao, Ying Qu, Changshun Huang, Chengzhilin Li, Wenyu Zhang, Xinyu Wang, Wenlong Duan, Qingbin He, Yachao Zhang, Jianwei Jiao, Runxiao Zheng

**Affiliations:** a Shandong Provincial Hospital Affiliated to Shandong First Medical University, Medical Science and Technology Innovation Center, Jinan, 250117, China; b Shandong Hongkui Medical Laboratory Co., Ltd., Jinan, 271100, China; c State Key Laboratory of Stem Cell and Reproductive Biology, Institute of Zoology, Chinese Academy of Sciences, Beijing, 100101, China

**Keywords:** Sepsis, Neutrophil, NAD^+^, ROS, Inflammation

## Abstract

Systemic inflammation, excessive reactive oxygen species (ROS) and mitochondrial impairment are the main cause of multi-organ dysfunction syndrome in sepsis. Nevertheless, the pharmaceuticals currently in development focus solely on a single mechanism of disease, which is evidently inadequate. Herein, a precision nanodrug delivery system (MSe-NAD^+^/Nes) has been designed, incorporating mesoporous selenium nanozymes (MSe NPs) and leveraging a neutrophil-targeting strategy, to accomplish accurate delivery and mitigate inflammation. Upon reaching the inflammatory region, MSe NPs destroys selenium bonds and releases NAD^+^ under the action of ROS, which in turn supplements the NAD^+^ pool and promotes the recovery of mitochondrial function. Moreover, MSe NPs are capable of efficiently eliminating ROS by mimicking the activity of glutathione peroxidase (GPx), thus preventing the activation of the NLRP3 inflammasome. *In vivo* administration has indicated that MSe-NAD^+^/Nes efficiently alleviates organ oxidative stress, restores ATP levels, attenuates systemic hyperinflammation, and facilitates rapid organ repair. This study presents a potential modality of inflammation remission via ROS scavenging and mitochondrial repairment for the reliable and safe therapy of sepsis.

## Graphical abstract

**Figure:**
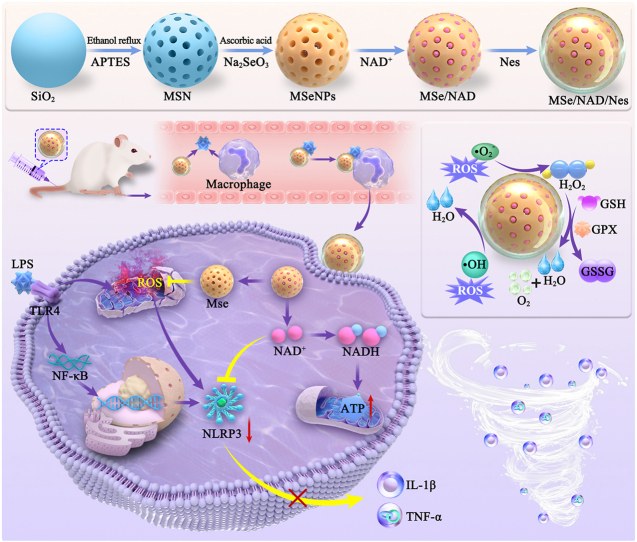

## 1 Introduction

Sepsis is a life-threatening disorder originated from dysregulated host response to bacterial, fungal, or viral infections, with bacterial infections being the predominant common. Globally, sepsis affects approximately 19 million individuals each year, with septic shock resulting in death rates of 35–40 % despite the administration of the best available therapeutic interventions [1]. In sepsis, upregulated NOD-like receptor thermal protein domain associated protein 3 (NLRP3) mRNA transcription as a result of bacterial infections gives rise to the overproduction of deleterious reactive oxygen species (ROS), which further exacerbates additional tissue inflammation and escalates ROS generation [2]. Excessive ROS inflicts oxidative damage on biomacromolecules, including lipids, proteins, and DNA, resulting in the impairment of organelles structural and functional integrity [3]. Mitochondria, being the primary source of ROS within cells, are particularly susceptible to ROS-induced damage under conditions of oxidative stress, making them the most vulnerable subcellular components to such assaults [4,5]. Hence, enhancing the restoration of mitochondrial functionality and the elimination of ROS to suppress the activation of the NLRP3 inflammasome is of significant importance in the management of sepsis.

The occurrence of multiple organ failure, inflammation, and mitochondrial dysfunction during sepsis are significantly correlated with depletion of nicotinamide adenine dinucleotide (NAD^+^) [6], which serves as a pivotal cofactor within the mitochondrial respiratory chain and plays a significant role in the regulation of cellular metabolism [7]. A recent study indicated that the administration of exceedingly high levels of NAD^+^ prior to injection exhibited a notably lower mortality rate to sepsis [6]. Comprehensive research has demonstrated that the delivery of NAD^+^ via calcium phosphate (CaP) NP or hollow mesoporous polydopamine (HMPDA) has yielded promising outcomes in the management of sepsis [8]. Moreover, supplementation with NAD^+^ has been verified to block both the classical and non-classical NLRP3 inflammasome pathways [9]. The cumulative evidence implies that administration of exogenous NAD^+^ to restore mitochondrial function and suppress the NLRP3 inflammasome pathways could represent an optimal therapeutic approach for sepsis treatment [10]. Nevertheless, NAD^+^, being a negatively charged hydrophilic small molecule, exhibits limited permeability through cellular membranes [11].

In contrast to other inflammatory diseases, the primary pathological feature of sepsis is the presence of the dynamic and intricate systemic inflammatory state, resulting in a dearth of tailored treatment options [12]. Neutrophils, which are among the most prevalent leukocytes in peripheral blood, play a crucial role in combating foreign pathogens by phagocytosis, degranulation and the formation of neutrophil extracellular traps (NETs) at sites of inflammation [13]. Neutrophils exhibit a characteristic chemotactic response to inflammation, enabling them to rapidly migrate to and accumulate in inflamed tissues [14]. Additionally, increasing evidence has shown that neutrophils play roles in suppressing the immune response and protecting the host, which are essential for tissue repair [15]. Depleting neutrophils in experimental animal models led to an exacerbation of lipopolysaccharide (LPS)-induced sepsis, highlighting the role of neutrophils in achieving optimal host protection [16]. And neutrophil membranes can also interact with inflammatory cytokines, thereby neutralizing the inflammatory response [17]. Upon stimulation by inflammatory cytokines, neutrophils are capable of generating NETs (including DNA and associated proteolytic enzymes) through the extrusion of their intracellular components, which process facilitates the release of cargoes from cellular barriers [18]. Meanwhile, neutrophils that have migrated to the site of injury may undergo diverse modes of cellular demise (including apoptosis, necrosis, and NETs). These spent neutrophils are thereafter engulfed and eradicated by macrophages, thus maintaining tissue homeostasis and mitigating further inflammatory damage [19]. Therefore, take advantage of the migratory and targeting capabilities of neutrophils, allowing NAD^+^ for precise and targeted drug delivery, thereby mitigating inflammation and potentially lowering sepsis-related mortality could serve as a significant strategy for targeting inflammatory foci in sepsis.

**Fig. 1:**
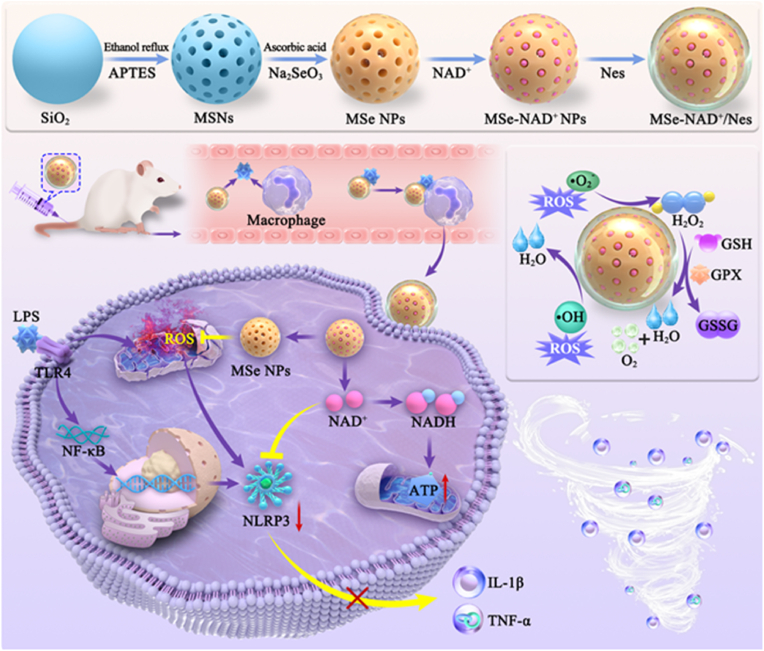
Schematic diagram of MSe-NAD^+^/Nes nanoplatforms as neutralize ROS and attenuate the inflammatory response for sepsis. Leveraging a neutrophil-targeting strategy to accomplish accurate delivery and mitigate inflammation. Upon reaching the inflammatory region, MSe NPs destroys selenium bonds and releases NAD^+^ under the action of ROS, which enhance mitochondrial homeostasis and cellular energy supply by effectively delivering NAD^+^ into cells. Meanwhile, MSe NPs are capable of efficiently eliminating ROS by mimicking the activity of glutathione peroxidase (GPx), thus preventing the activation of the NLRP3 inflammasome activation and attenuate the level of inflammatory factors, reduce the production of inflammatory factor storms, alleviate the condition of sepsis and effectively improve organ dysfunction.

Herein, we have developed an inflammation-targeting nanoplatform, denoted as MSe-NAD^+^/Nes, in which employs mesoporous selenium nanoparticles (MSe NPs) to deliver NAD^+^ and are subsequently internalized into neutrophils. As illustrated in Fig. 1, neutrophils are inflammation-responsive cells with pronounced affinity to the injured site. Then, MSe NPs efficiently eliminate ROS by mimicking the activity of glutathione peroxidase (GPx) at inflammatory environment and liberate NAD^+^ upon the disruption of selenium bonds within ROS stimuli [20]. The replenishment of NAD^+^ pool promoted the recovery of mitochondrial function and further alleviated NLRP3 inflammasome-mediated sepsis by ROS elimination. At the cellular level, MSe-NAD^+^/Nes can scavenge excessive ROS, restore mitochondrial function, and inhibit the NLRP3 inflammasome in inflammatory cells (RAW264.7). The treatment of MSe-NAD^+^/Nes *in vivo* with a sepsis model exhibited a remarkable therapeutic effect on the degree of damage to the infected organs throughout the body, oxidative stress, and inflammation suppression. This objective of study was to devise a strategy that combats inflammation by focusing on mitochondrial restoration and ROS elimination in sepsis treatment, which introduced novel concepts for the secure delivery of NAD^+^, with the goal of facilitating its clinical translation and practical application.

## 2 Experimental section

### 2.1 Materials

Hexadecyl trimethyl ammonium bromide (CTAB), 3-Aminopropyltriethoxysilane (APTES) and Ascorbic acid (99 %) were purchased from Shanghai Aladdin Biochemical Technology Co., Ltd. Sodium selenite (Na_2_SeO_3_,99 %), tetraethyl orthosilicate (TEOS), NAD^+^, and lipopolysaccharides were procured from Sigma Aldrich Trading Co., Ltd. Other reagents and solvents were purchased from Beijing Chemical Plant and used as received unless otherwise specified.

### 2.2 Synthesis of MSe NPs

CTAB (0.265 g) was dissolved in ethanol (7.5 mL) and deionized water (65 mL) at pH 9–10, TEOS was added while stirring, and the reaction was raised to 60 °C for 12 h. After centrifugation, the precipitate was washed three times with ethanol. After refluxing in 30 mL of ethanol (containing 1 mL of HCl) for 3 h, the product was washed with ethanol and added to APTES to obtain the aminated mesoporous silica (MSNs). MSNs (2 mg/mL) was prepared, sodium selenite (5 mg/mL) was added and stirred thoroughly for 30 min, ascorbic acid was added slowly and the reaction was carried out for 3 h. After the reaction, 200 μL of hydrofluoric acid (HF) was added to obtain MSe NPs.

### 2.3 NAD ^+^ loading to obtain MSe-NAD^+^ NPs

NAD^+^ dissolved in ultrapure water was added into the MSe NPs solution to ultrasound to obtain a clear solution, and was further stirred for 2 h. After 2 h of reaction at room temperature, most of un-loaded free NAD^+^ was precipitated and discarded by centrifugation at 2000 rpm for 5 min. NPs containing NAD^+^ (MSe-NAD^+^ NPs) were centrifuge (13 000 rpm, 10 min), washed in ultrapure water, and centrifuged again for 10 min. NAD^+^ loading capacity (LC) was calculated as following: [loading NAD^+^]/[MSe NPs] (*w/w*) %.

### 2.4 Separation and characterization of neutrophils from bone marrow

Mature neutrophils were isolated from the bone marrow through the previous method [21]. The femurs and tibias of mice were isolated, and the muscles, tendons and sinew were removed and immersed in phosphate-buffered saline (PBS) buffer solution. After flushing the bone marrow with 1640 medium, the precipitate was centrifuged and lysed after being resuspended with 5 mL of red blood cell lysis buffer. The resultant precipitate was resuspended with 1640 medium and added to a percoll mixture solution consisting of 55 %, 65 %, 75 % (*v/v*) percoll in PBS solution. After centrifugation at 1000*g* for 30 min, 65 % and 55 % of the separated interface of mature neutrophils were recovered and washed three times with PBS.

The purity was determined by immunofluorescence double staining with APC-coupled Ly-6G antibody and CD11b antibody coupled with FITC (BioLegend, USA) and detected via flow cytometry (Invitrogen, USA). Wright-Giemsa staining (Solarbio, China) was employed to detect neutrophil morphology.

### 2.5 Preparation and characterization of MSe-NAD^+^/Nes

The neutrophils (1 × 10^6^ cells/mL) were obtained from bone marrow were co-incubated with MSe-NAD^+^ NPs in RPMI 1640 medium at 37 °C for 1 h. The mixture was then centrifuged (1000 rpm, 5 min) and washed three times with PBS to obtain MSe-NAD^+^/Nes. For the preparation of Nes conjugated with fluorescent nanoparticles, Nes (1 × 10^6^ cells/mL) were incubated together with Rhodamine B-labeled MSe nanoparticles and FITC-labeled NAD^+^ at 37 °C for 1 h. Hoechst 33342 for neutrophil nuclear staining, fixed with 4 % paraformaldehyde (PFA), and used for confocal laser scanning microscope (CLSM) (ZEISS, LSM 880, Germany) analysis.

### 2.6 Characterization

Transmission electron microscopic (TEM) (Regulus 8100, Japan) was employed to analyze morphology, EDS (TESCAN MIRA LMS, Czech Republic) was utilized to analyze elemental composition, Brunauer-Emmett-Teller (BET) Surface Area Analysis (Micromeritics ASAP 2460, USA) was used to obtain information on specific surface area and pore size, and Dynamic Light Scattering and Zeta Potential (Nano ZS90, UK) was used to measure particle size and potential, Fourier Transform Infrared Spectroscopy (FTIR) (Thermo Fisher Scientific Nicolet iS20, USA) was used to employed to analyze characteristic functional group information.

### 2.7 *In vitro* NAD ^+^ release studies

*In vitro* NAD^+^ release was studied using the Ultraviolet–visible spectroscopy (*UV–vis*) spectrophotometry. 10 mg MSe-NAD^+^ NPs dissolve in 10 mL medium (PBS buffer, pH 7.4, containing 0/100/200 μM H_2_O_2_ as the dissolution medium) under horizontal shaking (60 rpm) at 37 °C. After 0.5, 1, 2, 4, 6, 8, 12, 24, 36 and 48 h, collected the whole medium and replaced with fresh medium. The experiment was performed parallel in triplicate.

### 2.8 GPx like activity assay

GPx activity of MSNs, NAD^+^, MSe NPs or MSe-NAD^+^ NPs was determined by measuring the absorbance of Nicotinamide adenine nucleotide phosphate (NADPH) at 340 nm monitor the glutathione teductases (GR)-couple reaction. Briefly, glutathione (GSH, 2 mM), H_2_O_2_ (200 μM), GR (2U), NADPH (0.4 mM), and MSNs, NAD^+^, MSe NPs or MSe-NAD^+^ NPs (40 μg/mL) were composed in 1 mL of PBS solution (100 mM) at pH 7.4. Natural GPx was utilized as a control group and reacted at different temperatures and pH for 10 min to investigate the effect of different factors on GPx-like activity.

### 2.9 ROS scavenging capability

The ·O_2_^−^ scavenging capacity of MSNs, NAD^+^, MSe NPs or MSe-NAD^+^ NPs was evaluated by monitoring the fluorescence curve using dihydroethidium (DHE) probe method. Xanthine (X) solution was prepared with NaOH (10 mM). 46 μL X (0.6 mM) and 4 μL xanthine oxidase (XO, 0.05 U mL^−1^) were added to 1 mL of PBS buffer, and the reaction was conducted at 37 °C for 40 min. After the reaction was completed, MSNs, NAD^+^, MSe NPs or MSe-NAD^+^ NPs were added to the reaction for 40 min. At last, DHE probe was added and incubated for 40 min to detect the fluorescence intensity.

The ·OH scavenging capacity of MSNs, NAD^+^, MSe NPs or MSe-NAD^+^ NPs was evaluated by *UV–vis* spectrophotometric detection of absorbance at 510 nm. FeSO_4_·7H_2_O (7.5 mM) was configured with deionized water at pH 3–4, and H_2_O_2_ (9.79 mM) was mixed and stirred with FeSO_4_·7H_2_O solution (*v:v* = 1:1) for 10 min. Subsequently, MSNs, NAD^+^, MSe NPs or MSe-NAD^+^ NPs were added to react for 60 min and then salicylic acid (SA) solution (1.8 mM) was added to react for 15 min, and the absorbance was measured at 510 nm by *UV–vis*.

The elimination rate of NPs is calculated according to the following formula:

$$Elimination\;\left(\%\right)=\frac{{A_{0}-A}_{S}}{A_{0}}\times 100\%$$

*A*_*0*_ and *A*_*S*_ are the absorbance and fluorescence values in the absence and presence of NPs, respectively.

### 2.10 Electro-catalytic activity of ROS

An electrochemical analyzer (CHI660D, Shanghai) was employed to evaluate the current density of MSNs, NAD^+^, MSe NPs or MSe-NAD^+^ NPs reacting with ROS solution and measure the cyclic voltammetry (CV) curves. A platinum wire and a saturated calomel electrodes (SCE) chosen as the counter electrode and reference electrode, and the MSNs, NAD^+^, MSe NPs or MSe-NAD^+^ NPs (2 mg/mL,10 μL) were dropped on the glassy-carbon electrode (GCE) and waited for natural drying. Subsequently, Nafion solution (3 μL) was added and deposited on the working electrode to prevent NPs drop. The GCE was soaked in ROS solutions (1 mM H_2_O_2_) and the CV curve was recorded and plotted at a scanning rate of 50 mV/s and a potential range of 0.5 V–2.0 V.

### 2.11 *In vitro* toxicity assessment

The RAW264.7 cells were purchased from the Shanghai Cell Bank and were cultivated in 1640 medium supplemented with 10 % fetal bovine serum (Gibco, USA) and 1 % penicillin/streptomycin solution (Hyclone, USA). The incubation condition was maintained at 37 °C in a humidified 5 % CO_2_ atmosphere.

RAW264.7 cells were cultured in 96-well plates for overnight. MSe NPs, NAD^+^ and MSe-NAD^+^ NPs were diluted to final concentrations of 0, 12.5, 25, 50, 100 and 200 μg/mL in cell medium, respectively, and incubated for 24 h. The 3-(4,5-dimethylthiazolyl-2)-2,5- diphenyltetrazolium bromide (MTT) was added to the culture for 3.5 h, and the microplate reader was used for detection. Then cells were stained with propidium iodide (PI) and calcein acetoxymethyl ester (AM) for 30 min and observed by a fluorescence microscope.

### 2.12 Cell viability and ATP level assay

RAW264.7 was cultivated in 96-well plate and cultured the cells to be fully adhered before being incubated with MSe/Nes, NAD^+^/Nes, and MSe/NAD^+^/Nes (equivalent to 25 μg/mL MSe NPs or 6.5 μg/mL NAD^+^), together with LPS (100 ng/mL) for 24 h. Then, the cells were washed three times with PBS, and MTT solution was added, the absorbance at 490 nm was measured using a microplate reader. Similarly, cells were collected to detect cellular ATP levels using an ATP chemiluminescence kit (Beyotime Biotechnology, Catalog No. S0027) in accordance with the manufacture's protocol.

### 2.13 Detection of NAD ^+^ levels

RAW264.7 cells were inoculated in 24-well plates and treated with LPS (100 ng/mL) with different treatment of PBS, MSe/Nes, NAD^+^/Nes, and MSe/NAD^+^/Nes. After 1 million cells were washed three times with PBS, add 200 μL of NAD^+^/NADH extraction solution. Centrifuged at 12 000 *g*, 4 °C for 5–10 min, and the supernatant was taken as the sample to be assayed according to the manufacturer's instructions (Catalog No. S0176S, Beyotime).

### 2.14 Detection of intracellular ROS levels and mitochondrial membrane potentials

RAW264.7 cells were inoculated in 6-well plates at a density of 1 × 10^5^ cells/well. The cells were treated with same way as before then washed three times with PBS, and then stained with 2,7-dichlorodihydrofluorescein diacetate (DCF) (Catalog No. D52C2, Cell signaling) for 30 min after which intracellular DCF fluorescence intensity was quantified by flow cytometry. Similarly, images were obtained by fluorescence microscopy after incubation with MitoSOX Red (Catalog No.AF7281, Beyotime) for 10 min at 37 °C. The excitation wavelength of MitoSOX Red spectra was 510 nm and the emission wavelength was 580 nm. JC-1 (Catalog No. G2083, Servicebio) was also used for incubation at 37 °C for 20 min, staining of nuclei with Hoechst, and image acquisition under a fluorescence microscope (OLYMPUS TH4-200, ZEISS).

### 2.15 NF-κB p65 nuclear translocation

Cells were inoculated in confocal dishes and cultured overnight. After incubation with PBS, MSe/Nes, NAD^+^/Nes, and MSe/NAD^+^/Nes (equivalent to 25 μg/mL MSe NPs or 6.5 μg/mL NAD^+^), the cells were treated with LPS (100 ng/mL). Then, 4 % paraformaldehyde was employed to fix the cells for 30 min at room temperature, followed by 0.5 % TritonX-100 permeating agent for 15 min. Subsequently, 10 % BSA was blocked for closure for 1 h at room temperature and the cells were incubated with NF-κB p65 antibody (Proteintech, Wuhan) overnight. Afterwards, the cells were incubated with Alexa Fluor 488-labeled secondary antibody for 1 h at room temperature. Then stained with DAPI for 10 min at room temperature, washed with PBST, and p65 nuclear translocation by CLSM to observed (Cell discoverer 7, Germany).

### 2.16 Western blot analysis

After incubating cells with different treatment of PBS, MSe/Nes, NAD^+^/Nes and MSe/NAD^+^/Nes, using a protease inhibitor-containing RIPA buffer to lyse the cells and prepare protein samples. Protein samples were loaded onto SDS-PAGE gel for protein isolation, which were subsequently transferred to polyvinylidene membranes (PVDF). After 1 h of containment in 5 % skim milk powder, the membrane was incubated with TLR4 (1:1000 dilution, Abcam), NF-κB p65 (1:1000, Proteintech), Phospho-NF-κB p65 (1:1000, Cell Signaling Technology), NLRP3 (1:1000 dilution, Abcam) and β-actin (1:5000 dilution, Proteintech) antibody for 2 h at room temperature. Subsequently, TBST washes were conducted three times for 10 min each followed by conjugation with HRP-labeled secondary antibody for 1 h at room temperature, and bands were detected using ECL luminescent solution (Catalog No. BL520A, Biosharp).

### 2.17 Measurement of inflammation-related cytokine levels

RAW264.7 cells were inoculated in 24-well plates to overnight and then incubated with LPS (100 ng/mL) together with PBS, MSe/Nes, NAD^+^/Nes, and MSe/NAD^+^/Nes (equivalent to 25 μg/mL MSe NPs or 6.5 μg/mL NAD^+^). The IL-1β (Catalog NO.430904, Biolegend) and TNF-α (Catalog NO.305457-009, Invitrogen) levels in the cell culture medium were tested by ELISA kit, respectively. The levels of inflammatory factors were calculated by detecting the difference in absorbance at 450 nm and 570 nm using an enzyme marker.

### 2.18 LPS-induced sepsis animal model

BALB/c mice (8–12 weeks, female) were purchased from the Jinan Pengyue Experimental animal Breeding Co., Ltd, with license number: SCXK(Lu)20220006. All the animals were handled in accordance with the Guide for Care and Use of Laboratory Animals, which was approved by the Animal Experimentation Ethics Committee of Shandong First Medical University. Based on the previous model [10], mice were utilized to establish a systemic inflammation model of sepsis induction by tail vein injection of LPS (15 mg/kg). After 1 h, mice were randomly grouped and intravenously administered with 100 μL of MSe-NAD^+^/Nes (equivalent to 2 mg/kg MSe NPs and 0.5 mg/kg NAD^+^). The survival state of the mice was monitored throughout the experimental period, and the body weight and survival rate of the mice were recorded over time.

Blood was collected from the eye sockets after LPS induction 12 h, serum levels of pro-inflammatory cytokines (TNF-α, IL-6 and IL-1β) were determined. After 24 h of LPS induction, mice were sacrificed. (1) Liver (ALT, AST) and kidney (BUN, CREA, UA) parameters were measured concurrently to assess the degree of inflammatory response. (2) The collection of heart, liver, spleen, lung and kidney of mice was carried out, and the organ weights were recorded as wet weights. Dry weights were recorded after drying in an oven at 60 °C for 24 h. The wet-dry ratio was recorded using the following formula: (3) The heart, liver, spleen, lung and kidney were fixed with 4 % paraformaldehyde, sectioned through dehydration, paraffin-embedded and subjected to histological examination by H&E staining. (4) Different organs or tissues were collected, weighed, homogenization and lysed for ATP quantification with a luminescent ATP assay kit (Catalog No. S0026, Beyotime).

$$Wet/Dry\;Ratio\left(\%\right)=\frac{Wet\;weight\left(mg\right)}{Dry\;weight\left(mg\right)}\times 100\%$$

### 2.19 *In vivo* biocompatibility and distribution

The hemolysis assay was employed to test the blood compatibility of the MSe-NAD^+^ NPs [22]. Specifically, mouse erythrocytes were obtained and resuspended in PBS, and then different concentrations of MSe-NAD^+^ NPs were added. Deionized water diluted erythrocytes were utilized as a positive control and PBS as a negative control, and the absorbance was measured by reacting at 37 °C for 3 h. The hemolysis rate was calculated according with the following formula:

$$Hemolysis\;rate\left(\%\right)=\frac{A_{S}-A_{n}}{A_{P}-A_{n}}\times 100\%$$

Where *As* is the absorbance of the test sample, *An* and *Ap* are the absorbances of the negative and positive controls respectively.

After co-incubating MSe-NAD^+^/Nes with Cy5 fluorescent dye for 12 h, Cy5 probes loaded with MSe-NAD^+^/Nes were prepared. The mice were divided into 2 groups: 1) Healthy mice + MSe-NAD^+^/Nes; 2) LPS-induced sepsis mice + MSe-NAD^+^/Nes. Cy5-MSe-NAD^+^/Nes was injected into healthy or septic mice via the tail vein. The mice were sacrificed 24 h later, the organs were dissected and fluorescence imaging was performed. Similarly, after LPS-induced sepsis was established, the mice were injected MSe-NAD^+^/Nes by intravenous injection. Collected the heart, liver, spleen, lung and kidney dissolved in aqua regia, and the distribution of Selenium content in each organ was examined using ICP-MS (Aquion RFIC-iCAP TQ).

### 2.20 *Invivo* antioxidant performance

The ability of PBS, MSe/Nes, NAD^+^/Nes and MSe/NAD^+^/Nes to clear ROS levels in various organs was determined by DHE fluorescent probe. Fresh heart, liver, spleen, lung and kidney tissue was fixed and sectioned. DHE staining and nuclear DAPI staining were performed according to the protocol of the reagent manufacturer, and the fluorescence intensity was observed under an inverted fluorescence microscope.

### 2.21 Immunohistochemical staining

The ability of PBS, MSe/Nes, NAD^+^/Nes and MSe/NAD^+^/Nes to clear NLRP3 protein levels in organs was determined by immunohistochemistry. Collected organs were embedded and sectioned for fixation, 10 mM sodium citrate (pH 6.0) buffer for antigen repair, BSA for serum closure, incubated with NLRP3 primary antibody overnight, added secondary antibody followed by staining, dehydrated and sealed. The samples were observed under an inverted fluorescence microscope and analyzed for positive area using Image J software.

### 2.22 Statistical analysis

Statistical analysis was using GraphPad Prism software. The numerical comparison was performed by a one-way ANOVA test followed by Tukey's post hoc comparison for analyzing the differences between the differences two groups. The results are shown as the mean ± SD, and the significance level is defined as *p* < 0.05 (95 % confidence level).

## 3 Results and discussion

### 3.1 Synthesis and characterization of MSe-NAD^+^/Nes

**Fig. 2:**
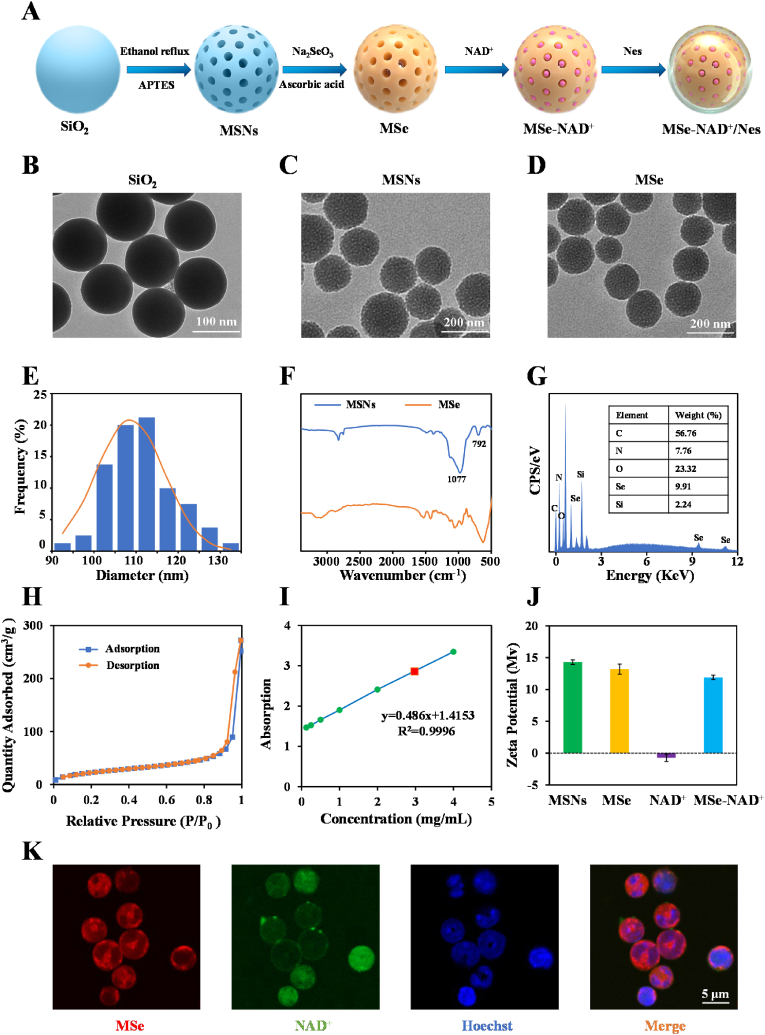
Synthesis and characterization of MSe-NAD^+^/Nes. Scheme of the preparation of MSe-NAD^+^/Nes (A); The TEM image of SiO_2_ (B), MSNs (C), MSe NPs (D); The statistical graph of particle size distribution of TEM image of MSe NPs (E); FTIR spectrum of MSNs and MSe NPs (F); The elements quantitative analysis of MSe NPs by EDS elemental analysis (G); Nitrogen adsorption desorption isotherms for MSe NPs (H); *UV–vis* spectroscopy calibration curve for measuring NAD ^+^ loading efficiency (I); Zeta potential of MSNs, MSe NPs, NAD^+^, MSe-NAD^+^ NPs during the preparation process (J); CLSM images of MSe-NAD^+^/Nes after culturing at 37 °C. The nuclei of neutrophils were stained with Hoechst 33342 (Blue), MSe NPs were stained with rhodamine B (Red), and NAD^+^ was labeled with FITC (Green) (K).

MSe NPs were fabricated utilizing the “hard template” method [23]. Firstly, we fore-synthesized the MSNs as “hard template”. Subsequently, Na_2_SeO_3_ was deposited to the surface of MSNs, followed by the addition of ascorbic acid to selectively etch the MSNs to yield MSe NPs (Fig. 2A). As illustrated in Fig. 2B, the SiO_2_ showed uniform morphology and similarly spherical structures. TEM images (Fig. 2C) showed that the synthesized MSNs possess a large pore size, high dispersity and uniform spherical morphology. MSe NPs exhibited a uniform spherical shape and were monodisperse in Fig. 2D. The TEM particle size distribution diagram (Fig. 2E) shows that the diameter of MSe NPs is 112.5 ± 7.8 nm. The FTIR spectra of the MSNs revealed characteristic stretching vibrations at 1077 cm^−1^ and bending vibrations at 792 cm^−1^, corresponding to the Si-O-Si bonds, consistent with prior studies [24]. In MSe NPs, the absorption peak associated with the Si-O-Si bond at 1077 cm^−1^ is markedly diminished, suggesting near-complete removal of the MSNs template, as depicted in Fig. 2F. Moreover, Energy-dispersive X-ray spectroscopy (EDS) (Fig. 2G) shows that the selenium and silicon contents in MSe NPs are 9.9 % and 2.2 %, respectively.

According to the BET analysis, the surface area of MSe NPs was determined to be 319 m^2^/g with a pore size of 19.3 nm (Fig. 2H and S1), which facilitates the exposure of highly active sites for scavenging ROS and drug delivery [25]. The loading rate of NAD^+^ was determined by calculating the difference between the initial amount of NAD^+^ used and the remaining NAD^+^ in the supernatant, resulting in a loading rate of 25.5 %. It is well established that the zeta potential of nanoparticles is closely associated with their surface charge (Fig. 2I). Silica samples typically exhibit negative zeta potential values due to the presence of numerous silanol groups on their surfaces [26]. However, the incorporation of amino groups resulted in a zeta potential of 14.3 ± 0.4 mV, which indicated the successful synthesis of MSNs (Fig. 2J). SeO_3_^2−^ adsorbed to the amido group on the MSNs surface through electrostatic interaction, resulting in a decrease in the MSe NPs potential. When NAD^+^ is loaded into MSe NPs as a negatively charged hydrophilic small molecule drug, the potential of MSe-NAD^+^ NPs is lower 1.3 mV than MSe NPs, which also confirms the successful loading of NAD^+^ [10]. Furthermore, the stability of MSe-NAD^+^ NPs when dispersed in water and culture medium was examined, demonstrated that the particle dimensions maintained their stability for one week (Fig. S2).

Furthermore, to improve the precision targeting of sepsis hotspots, mature neutrophils are employed for the delivery of MSe-NAD^+^ NPs. Neutrophils have an active inflammatory tendency and is capable of crossing dense boundaries to penetrate deeper into the tissues [27,28]. However, the individual neutrophil cell membrane lack active migration ability and rely on the blood circulation to passively reach the lesion area, with limited targeting ability for deep tissues or tissues in areas with low blood flow [29]. Early sepsis mortality is caused by an acute, deleterious pro-inflammatory response. Active drug delivery by neutrophils targeting the inflammatory site is the key to eliminating pathogenic microorganisms of sepsis and controlling the progression of the disease. Mouse bone marrow-derived neutrophils were isolated and labeled using a double staining protocol with FITC-conjugated anti-mouse CD11b and APC-conjugated anti-mouse Ly6G antibodies. Flow cytometry analysis revealed that the purity of the extracted neutrophils was up to 93.2 % (Fig. S3A). In addition, the neutrophils were subjected to Giemsa-Wright nuclear staining, which revealed that the morphology of the extracted neutrophils exhibited the characteristic bilobed shape (Fig. S3B). The purified neutrophils were incubated with MSe-NAD^+^ NPs and incubation time of 1 h to obtain MSe-NAD^+^/Nes. The migratory function of MSe-NAD^+^/Nes was tested *in vitro* using a transwell migration assay. The results of *in vitro* transwell experiments showed that natural neutrophils had the best inflammatory tendency, followed by MSe-NAD^+^/Nes encapsulated by neutrophils (Fig. S4A).

CLSM was applied to validate the successful construction of MSe-NAD^+^/Nes by the fluorescence co-localization of Rhodamine B-labeled MSe NPs and FITC-labled NAD^+^. As shown in Fig. 2K, the red fluorescence of MSe NPs and the green fluorescence of NAD^+^ are uniformly distributed within the neutrophils, which demonstrates that neutrophils can efficiently internalize MSe-NAD^+^ NPs. Moreover, neutrophil have good biocompatibility, low immunogenicity and extremely small systemic side effects [30]. However, nanomaterials loaded with cell membrane components in contact with a biological medium are rapidly comprehended by a number of protein molecules resulting in the formation of an NP-protein complex called protein corona (PC), which may affect cellular uptake [31]. To further investigate the uptake capacity of neutrophil-derived drugs by macrophages, we co-incubated Nes, MSe-NAD^+^/Nes, and MSe-NAD^+^ encapsulated by neutrophil cell membranes (mMSe-NAD^+^) with macrophages in the presence of IL-1β (10 ng/mL). Neutrophils were labeled with APC-Ly6G, macrophage nuclei were stained with Hoechst 33342 and MSe-NAD^+^/Nes NPs were labeled with FITC. The CLSM results showed that each group could be taken up by macrophages under the influence of IL-1β. However, the distribution of MSe-NAD^+^ in macrophages in the MSe-NAD^+^/Nes group was more uniform, which might be due to the prominent phagocytosis of neutrophils (Fig. S4B). Furthermore, we further conducted *in vitro* Transwell experiments to detect the fluorescence intensity of inferior lumen RAW264.7 cells at different periods. Cy5-labeled MSe-NAD^+^/Nes and mMSe-NAD^+^ were placed in the upper chamber of the Transwell system. At regular intervals, RAW 264.7 cells in the lower chamber of the Transwell were harvested. Subsequently, the cells in the lower chamber were gently rinsed 2–3 times with PBS to eliminate the un-internalized fluorescent drugs. After that, an appropriate volume of cell lysis buffer was added to the washed cells in the lower chamber. The lysate was then centrifuged, and the supernatant was collected for subsequent detection of fluorescence intensity. As the results showed, we found that MSe-NAD^+^/Nes has a stronger fluorescence intensity compared with mMSe-NAD^+^ under the same conditions, with the extension of incubation time, the signal of Cy5-MSe-NAD^+^/Nes located in macrophages gradually increased, indicating that MSe-NAD^+^ was successfully transferred into macrophages (Fig. S4C), which was similar with previous reports that macrophages could phagocyte the neutrophils completely [32].

### 3.2 ROS scavenging activity and NAD ^+^ release of MSe-NAD^+^/Nes

**Fig. 3:**
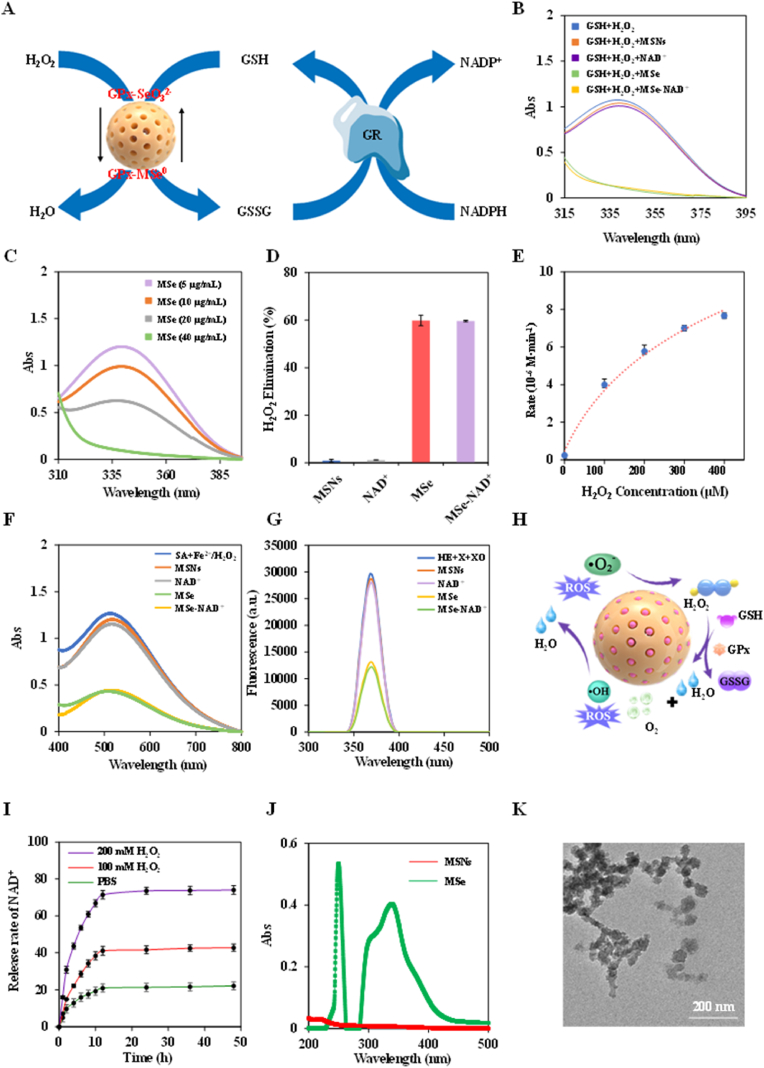
The Wide-ranging ROS scavenging activity of MSe-NAD^+^/Nes. Schematic diagram of MSe NPs simulating GPx in the elimination of H_2_O_2_ (A); The GPx-like activity of MSNs, NAD^+^, MSe NPs and MSe-NAD^+^ NPs (B); The GPx-like activity of different concentration MSe-NAD^+^ NPs (C); The H_2_O_2_ elimination efficiency of MSNs, NAD^+^, MSe NPs and MSe-NAD^+^ NPs (D); Michaelis-Menten plot with varying concentration of H_2_O_2_ (0–400 μM) for MSe NPs (E); *UV–vis* spectra of SA after reacting with ·OH, in presence of MSNs, NAD^+^, MSe NPs and MSe-NAD^+^ NPs respectively (F); Fluorescent spectra of HE after reacting with X and XO, in presence of MSNs, NAD^+^, MSe NPs and MSe-NAD^+^ NPs, respectively (G); Schematic diagram of the mechanism of multiple reactive oxygen species scavenging by MSe NPs (H); NAD^+^ release behaviors of MSe-NAD^+^ NPs. The release of NAD^+^ was observed with time in the presence of 0 μM, 100 μM, 200 μM H_2_O_2_ in PBS buffer at pH 7.4 (I); *UV–vis* spectra in spectra of MSNs solution and MSe NPs solution at room temperature (J); TEM image of MSe-NAD^+^ NPs responsiveness in simulated media (200 μM H_2_O_2_ in PBS buffer at pH 7.4) (K).

MSe NPs have been proven to efficiently eliminate H_2_O_2_, replicating the catalytic action of GPx, as illustrated in Fig. 3A. GPx promotes the oxidation reaction that transforms reduced GSH into glutathione disulfide (GSSG), while GR catalyzes the reduction of GSSG to regenerate GSH, with NADPH acting as an electron source. Thus, the activity of GPx is determined by monitoring the consumption of NADPH. Compared with MSNs and NAD^+^, both MSe NPs and MSe-NAD^+^ NPs demonstrated a reduction in absorbance at 340 nm, suggesting that they possess GPx-like activity in Fig. 3B. Moreover, GPx activity assays on varying concentrations of MSe-NAD^+^ NPs revealed a pronounced concentration-dependent response, as illustrated in Fig. 3C. The scavenging capabilities for H_2_O_2_ were measured and found to be 0.9 % for MSNs, 1.2 % for NAD^+^, 59.6 % for MSe NPs and 59.9 % MSe-NAD^+^ NPs as depicted in Fig. 3D. The Michaelis–Menten kinetic analysis for MSe-NAD^+^ NPs yielded a Vmax of 11.1 μM min^−1^ and Km is 178.48 μM, as presented in Fig. 3E.

Nevertheless, the primary challenge in utilizing natural enzymes for inflammation management is their poor stability, particularly given their sensitivity to environmental factors like pH and temperature [[33], [34], [35]]. To probe into the catalytic behaviors of MSe-NAD^+^ NPs, a comparative stability assessment was undertaken between natural GPx and MSe-NAD^+^ NPs across a range of acidic environments and temperatures (Fig. S5A and B). Under acidic conditions (pH 5.0) and elevated temperatures (60 °C), the catalytic efficacy of GPx was markedly diminished, with the H_2_O_2_ scavenging rate dropping to 14.3 % and 21 % as shown in Fig. S5C and S5D, respectively. In contrast, MSe-NAD ^+^ NPs maintained its H_2_O_2_ scavenging efficiency, unaffected by these conditions. The results indicated that MSe-NAD^+^ NPs exhibited potent GPx-like properties, positioning it as a viable candidate for efficiently scavenging ROS.

Besides H_2_O_2_, other ROS such as hydroxyl radicals (·OH) and superoxide anions (·O_2_^−^) also contribute to inflammation [[36], [37], [38]]. The interaction of Fe^2+^ with H_2_O_2_ through the Fenton reaction produces ·OH, capable of oxidizing salicylic acid (SA) and leading to a distinct absorption peak at 510 nm. This phenomenon can serve as a basis for evaluating the ·OH scavenging capacity of MSe-NAD^+^ NPs. As shown in Fig. 3F, the *UV–vis* absorbance intensity of MSe NPs and MSe-NAD^+^ NPs significantly decreases after reaction, whereas there is no significant change in MSNs and NAD^+^ groups. The ·OH scavenging efficiencies of MSNs, NAD^+^, MSe NPs and MSe-NAD^+^ NPs were calculated to be 22.1 % ± 3 %, 25.6 % ± 3.2 %, 70.9 % ± 3.4 % and 71.5 % ± 3.4 %, respectively (Fig. S6A). Moreover, the ·OH scavenging ability of MSe-NAD^+^ NPs at different concentrations was evaluated, and the results indicated a positive correlation between the concentration of MSe-NAD^+^ NPs and its ·OH scavenging efficacy (Fig. S6B). With the increase of MSe-NAD^+^ NPs concentration, the clearance rate of ·OH also increased gradually (Fig. S6C).

Subsequently, the DHE probe was used to evaluate the ·O_2_^−^ scavenging capacity of MSe-NAD^+^ NPs. As anticipated, a notable reduction in fluorescence intensity was observed for MSe-NAD^+^ NPs upon its reaction with the DHE probe, as illustrated in Fig. 3G. The ·O_2_^−^ clearance rates of MSNs, NAD^+^, MSe NPs, MSe-NAD^+^ NPs were 3.7 % ± 0.4 %, 7.5 % ± 1 %, 56 % ± 0.3 %, 59 % ± 0.5 %, respectively, as indicated in Fig. S6D. Additionally, MSe-NAD^+^ NPs demonstrated a pronounced concentration-dependent clearance of ·O_2_^−^, as evidenced in Fig. S6E–F. Besides, CV was employed to measure the current intensity of MSe NPs, which exhibited a more robust current intensity and a quicker electron transfer rate in the ROS solution than the unmodified GC electrode, with current intensities of 5.4 μA for MSe NPs and 6.7 μA for MSe-NAD^+^ NPs in Fig. S7. In short, the MSe-NAD^+^ NPs demonstrated a significant capacity to eliminate H_2_O_2_ by emulating GPx-like activity and possesses the ability to scavenge ·OH and ·O_2_^−^, making it a promising candidate as a broad-spectrum reactive oxygen species scavenger in Fig. 3H.

Oxidative stress and dysregulated inflammation lead to a detrimental loop in sepsis, which results in cytokine storms and multiorgan failure [39]. Therefore, we studied the NAD^+^ release behavior in PBS containing H_2_O_2_ to simulate the oxidative stress environment in sepsis. In high H_2_O_2_ concentration conditions (pH 7.4 PBS buffer containing 200 μM H_2_O_2_), NAD^+^ exhibited rapid drug release within the first 12 h, achieving a release rate of 71.5 % ± 2.3 % (Fig. 3I). Notably, under normal conditions (pH 7.4 PBS buffer without H_2_O_2_), the release rate was significantly slower, with only 20.9 % ± 1.8 % of the NAD^+^ released within the same period. These results demonstrate that MSe-NAD^+^ NPs possesses a robust ROS-responsive drug release capability. This phenomenon is attributed to the presence of Se-Se bonds in MSe NPs, which break in response to ROS, and this break may be involved in the inflammatory regulation process in sepsis (Fig. 3J) [40]. TEM images of MSe NPs after ROS exposure revealed significant morphological disruption, with particles becoming disordered and exhibiting reduced size (Fig. 3K). This observation provides compelling evidence for the ROS-induced cleavage of Se-Se bonds.

### 3.3 ROS scavenging and mitochondrial repairment of MSe-NAD^+^/Nes *in vitro*

**Fig. 4:**
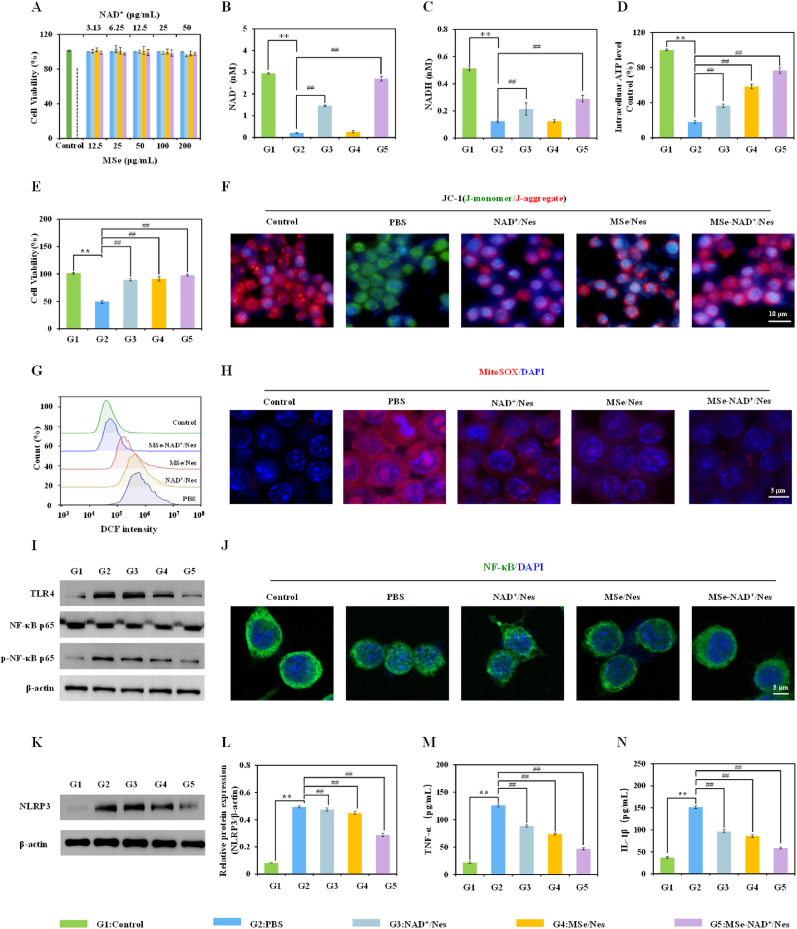
ROS scavenging and mitochondrial repairment of MSe-NAD^+^/Nes *in vitro*. RAW264.7 cells were incubated with different concentrations of control, MSe NPs, NAD^+^, and MSe-NAD^+^ NPs for 24 h, and the cell viability (A); The intracellular NAD^+^ (B) and NADH (C) level of RAW264.7 cells in NAD^+^/Nes, MSe/Nes, and MSe-NAD^+^/Nes in the LPS-induced energy consumption model, Raw264.7 cells not treated with LPS as a negative control; Quantification of intracellular ATP level (D) and cell viability (E) in an LPS-mediated energy depletion model; Fluorescence images of RAW264.7 cells stained with JC-1 were used to analyze the depolarization of mitochondrial membrane (F); Flow cytometry analysis of ROS in control, PBS, MSe/Nes, NAD^+^/Nes and MSe-NAD^+^/Nes treated RAW264.7 cells (G); MitoSOX staining analysis of RAW264.7 cells in control, PBS, MSe/Nes, NAD^+^/Nes and MSe-NAD^+^/Nes treatment groups (H); The expression level of TLR4, p-NF-κB, NF-κB protein detected by Western blot (I); The nuclear localization of NF-κB (p65) observed by CLSM (J); The expression level of NLRP3 protein detected by Western blot (K,L); The expression level of pro-inflammatory cytokines TNF-α (M)and IL-1β (N) detected by ELISA in the culture supernatant of RAW264.7 cells. The group without LPS stimulation was taken as the control group. RAW264.7 cells were treated with LPS (100 ng/mL) and then treated with PBS, MSe/Nes, NAD^+^/Nes and MSe/NAD^+^/Nes respectively (equivalent to 25 μg/mL MSe NPs or 6.5 μg/mL NAD^+^). G1 respresents Control group; G2 respresents PBS group; G3 respresents NAD^+^/Nes group; G4 respresents MSe/Nes group; G5 respresents MSe-NAD^+^/Nes group. Values are expressed as mean ± S.D. ∗∗*p* < 0.01, ∗*p* < 0.05 vs. control group, ^##^*p* < 0.01, ^#^*p* < 0.05 vs. PBS group.

Within the concentration range of 200 μg/mL, MSe NPs, NAD^+^, and MSe-NAD^+^ NPs had no obvious cytotoxic towards RAW264.7 cells (Fig. 4A), indicating good biocompatibility of MSe-NAD^+^ NPs. NAD^+^, a vital cofactor in cellular energy metabolism, participates in key pathways such as glycolysis and oxidative. When sepsis takes hold, the respiratory function within the affected cell becomes compromised, and the energy-metabolism pathway of the mitochondria is disrupted [41]. Therefore, sustaining NAD^+^ levels is essential to curb energy loss and avert cell damage associated with inflammation [42]. As illustrated in Fig. 4B and C, the content of NAD^+^ and NADH in Raw 264.7 cells induced by LPS decreased by 93% and 76% relative to control group. Compared with the PBS group, the level of NAD^+^ in the NAD^+^/Nes group, the MSe/Nes group, and the MSe-NAD^+^ group increased by 42.7%, 3.1%, and 84.7%, respectively. Under the induction of LPS, NAD^+^/Nes can significantly increase the content of intracellular NAD(H), which further confirmed that the NAD^+^ library could be “injected” through exogenous replenishment (Fig. 4C). Surprisingly, in the MSe-NAD^+^/Nes group, the intracellular NAD^+^ level approximated that of normal cells. This finding further validates that the NAD^+^ reservoir can not only be “filled” via exogenous supplementation but also stimulate the production of endogenous NAD^+^ by rejuvenating mitochondrial function. Moreover, the ratio of NAD^+^ to NADH serves as a crucial marker for assessing cellular metabolic and redox conditions. Notably, both MSe/Nes and MSe-NAD^+^/Nes have been found to substantially elevate this ratio in Fig. S8. In sepsis progression, cellular mitochondria dysfunction leads to a collapse of the redox state, making it challenging for cells to sustain their basic functions [41]. The results revealed that MSe-NAD^+^/Nes significantly boosted intracellular ATP supply in Fig. 4D, restored the mitochondrial redox balance and improved cell viability (Fig. 4E). Moreover, the results similarly to those of MTT were also obtained by the cell live/dead staining assay in Fig. S9.

in Raw 264.7 cells induced by LPS decreased by 93% and 76% relative to control group. Compared with the PBS group, the level of NAD^+^ in the NAD^+^/Nes group, the MSe/Nes group, and the MSe-NAD^+^ group increased by 42.7%, 3.1%, and 84.7%, respectively. Under the induction of LPS, NAD^+^/Nes can significantly increase the content of intracellular NAD(H), which further confirmed that the NAD^+^ library could be “injected” through exogenous replenishment (Fig. 4C). Surprisingly, in the MSe-NAD^+^/Nes group, the intracellular NAD^+^ level approximated that of normal cells. This finding further validates that the NAD^+^ reservoir can not only be “filled” via exogenous supplementation but also stimulate the production of endogenous NAD^+^ by rejuvenating mitochondrial function. Moreover, the ratio of NAD^+^ to NADH serves as a crucial marker for assessing cellular metabolic and redox conditions. Notably, both MSe/Nes and MSe-NAD^+^/Nes have been found to substantially elevate this ratio in Fig. S8. In sepsis progression, cellular mitochondria dysfunction leads to a collapse of the redox state, making it challenging for cells to sustain their basic functions [41]. The results revealed that MSe-NAD^+^/Nes significantly boosted intracellular ATP supply in Fig. 4D, restored the mitochondrial redox balance and improved cell viability (Fig. 4E). Moreover, the results similarly to those of MTT were also obtained by the cell live/dead staining assay in Fig. S9.

Sustained oxidative stress will lead to irreversible inflammatory damage, while initiating a cyclic process in which inflammatory response and ROS promote each other, further exacerbating inflammation in systemic sepsis [43,44]. In the relevant experiments for investigating activities, we have confirmed that MSe NPs possess broad-spectrum ROS scavenging capabilities. Meanwhile, NAD^+^ functions as a mediator for antiviral and anti-inflammatory responses [45]. Furthermore, extracellular vesicles released by circulating neutrophils facilitate their antithrombotic function by curbing the accumulation of reactive oxygen species in endothelial cells [16]. However, whether MSe-NAD^+^/Nes has the potential to ameliorate ROS production and inflammatory damage remains to be explored. The JC-1 probe is utilized to assess mitochondrial membrane potential, a key indicator of mitochondrial function. Compared with the PBS group, both MSe/Nes and MSe-NAD^+^/Nes significantly mitigated mitochondrial damage and restored mitochondrial function (Fig. 4F and S10). To further investigate the impact of oxidative stress on cells, the DCF probe was utilized to measure intracellular ROS levels. Flow cytometry results indicated that the PBS group exhibited the highest levels of DCF fluorescence, whereas treatment with MSe-NAD^+^/Nes led to a marked decrease in fluorescence values, suggesting a reduction in ROS levels following MSe-NAD^+^/Nes administration (Fig. 4G). Typically, DCFH-DA has no fluorescence, which can be oxidized by ROS to DCF with green fluorescence. Therefore, the generation and accumulation of intracellular ROS can be reflected according to the intensity of green fluorescence, respectively. As expected, the LPS-activated macrophages had obvious green fluorescence, indicating that LPS treatment could effectively trigger intracellular oxidative stress. Interestingly, after being treated with MSe-NAD^+^/Nes, they displayed a more excellent ROS scavenging capacity with an obvious fluorescence intensity decrease of DCF (Fig. S11). Additionally, MitoSOX Red is a mitochondria-specific fluorescent probe for living cells, primarily employed to measure mitochondrial ROS levels. Consistent with expectations, the PBS group exhibited the most intense red fluorescence. In contrast, treatments with MSe/Nes, NAD^+^/Nes, and MSe-NAD^+^/Nes resulted in a reduction of fluorescence intensity to different extents, with MSe-NAD^+^/Nes demonstrating the most potent scavenging effect on ROS overall (Fig. 4H and S12).

The macrophage membrane contains TLR4, which is an important receptor that triggers the LPS-activated TLR4/NF-κB/NLRP3 pathway to induce the inflammatory response [46,47]. In resting cells, NF-κB P65 is maintained in an inactive state within the cytoplasm, sequestered by its inhibitory protein [48]. When cells are stimulated by factors such as pathogen-associated molecular patterns and cytokines, NF-κB P65 mainly enters the cell nucleus. Activated NF-κB translocates to the nucleus and stimulates the release of inflammatory mediators, such as IL-1β, IL-6, and TNF-α [49]. The up-regulation and subsequent release of these pro-inflammatory cytokines play a pivotal role in the pathogenesis of sepsis, exacerbating the systemic inflammatory response and contributing to the progression of the disease. TLR4/NF-κB/NLRP3 pathway-modulated inflammation plays an imperative role in the process of sepsis. Hence, protein levels of TLR4, NF-κB p65, p-NF-κB p65 in the cell were examined by western blotting. As exhibited in Fig. 4I and S13, the protein abundance of TLR4 and p-NF-κB p65 was significantly upregulated in the PBS group (pretreated with LPS), when compared to the control group (without pretreated with LPS). However, supplement with MSe/Nes, NAD^+^/Nes and MSe-NAD^+^/Nes strikingly reversed these changes. NF-κB is one of the downstream core targets within the TLR4 signaling pathway. To further investigate the alleviation of inflammation, the activation of NF-κB after treatment with PBS, MSe/Nes, NAD^+^/Nes and MSe-NAD^+^/Nes was examined using CLSM. The results showed high expression of NF-κB (P65) activation transferred into the nucleus in the PBS group, however, P65 transfer into the nucleus was significantly reduced after MSe-NAD^+^/Nes treatment (Fig. 4J). Moreover, to assess the impact of MSe-NAD^+^/Nes on inflammasome activation, the expression of NLRP3 protein was examined using western blot analysis. The western blot analysis revealed a reduction in NLRP3 protein expression following treatments with NAD^+^/Nes, MSe/Nes and MSe-NAD^+^/Nes, as depicted in Fig. 4K and L. Besides, the expression levels of downstream inflammatory factors TNF-α and IL-1β were detected by Elisa assay. The production of these downstream pro-inflammatory cytokines was significantly suppressed, with MSe-NAD^+^/Nes exhibiting a stronger inhibitory effect on inflammation, as shown in Fig. 4M and N. In addition, the immunofluorescence results also showed LPS inducement notable enhanced the TNF-α and IL-1β expression of macrophage. Remarkably, MSe-NAD^+^/Nes treated LPS-activated macrophages presented an unobvious green fluorescence, the results demonstrated the excellent inflammation clearance capacity of MSe-NAD^+^/Nes in pro-inflammatory macrophages (Fig. S14A). As shown in Fig. S14B and C, LPS-stimulated macrophage in the MSe-NAD^+^/Nes group obviously reduced the proportion of fluorescence intensity, demonstrating that MSe-NAD^+^/Nes effectively improved the inflammatory environment. Overall, these results suggested that MSe-NAD^+^/Nes could mitigate inflammatory reactions by eliminating reactive oxygen species and restoring mitochondrial function.

### 3.4 Therapeutic efficacy of MSe-NAD^+^/Nes against LPS-induced sepsis *in vivo*

**Fig. 5:**
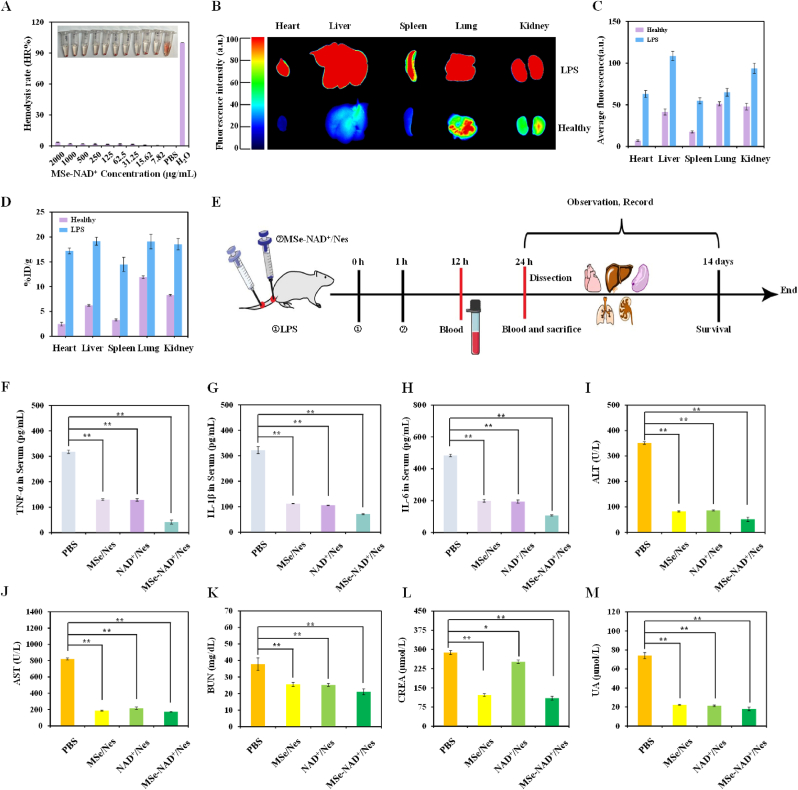
*In vivo* therapeutic effects of MSe-NAD^+^/Nes on sepsis model. Relative hemolysis rates of MSe-NAD^+^ NPs at different concentrations (A); Biodistribution of Cy5-labeled MSe-NAD^+^/Nes in healthy and septic mice after MSe-NAD^+^/Nes administration for 24 h (B); Quantitative analysis of the mean fluorescence intensity of the organ or tissue shown in the *ex vivo* images (C); ICP-MS analysis of selenium content in the dissected tissues of mice treated with MSe-NAD^+^/Nes after 24 h of administration (the data are expressed as the percentage of ID/g of the injected dose) (D); Experimental flowchart of the treatment process (E); Pro-inflammatory cytokine TNF-α (F), IL-1β (G), IL-6 (H) levels in the serum of septic mice receiving PBS, MSe/Nes, NAD^+^/Nes and MSe-NAD^+^/Nes treatments were measured after LPS injection 12 h; Serum ALT (I), AST (J), BUN (K), CREA (L) and UA (M) levels in each group; Values are expressed as mean ± S.D. ∗∗*p* < 0.01, ∗*p* < 0.05 vs. PBS group.

Biosafety is a major consideration in the use of NPs for clinical translational applications. To investigate *in vivo* hemolytic effects, hemolysis experiment was conducted with varying concentrations of MSe-NAD^+^ NPs treatment in Fig. 5A. The results indicated that MSe-NAD^+^ NPs groups did not exhibit significant hemolysis at concentrations up to 2 mg/mL, indicating their suitability for intravenous injection in mice. Furthermore, the biological distribution of MSe-NAD^+^/Nes was discovered with *in vivo* fluorescence imaging system. *Ex vivo* fluorescence images representing the biodistribution of Cy5-labeled MSe-NAD^+^/Nes in healthy and septic mice at 24 h after administration as showed in Fig. 5B and C. Compared with healthy mice, sepsis mice exhibited significantly stronger fluorescence across all major organs. This suggests that MSe-NAD^+^/Nes has an enhanced *in vivo* targeting ability towards inflammatory sites and accumulates rapidly within these areas, likely due to the chemotaxis of neutrophils towards inflamed tissues. In order to investigate the organ-specific uptake of MSe-NAD^+^/Nes in sepsis mice and healthy mice, the Selenium element content in various organs was quantified using ICP-MS (Fig. 5D). In healthy mice, Selenium elements were significantly enriched in lung and kidney tissues, likely due to the relatively larger particle size of MSe-NAD^+^/Nes and the critical metabolic functions performed by these organs. In sepsis mice, the enrichment of Selenium elements in various organs was significantly increased, which further indicated that MSe-NAD^+^/Nes has long-term circulation and efficient uptake ability in the inflammatory organs of sepsis mice, which is conducive to the treatment of sepsis.

Next, we injected LPS into the mouse tail vein induced endotoxemia model, and investigated the *in vivo* therapeutic properties of MSe-NAD^+^/Nes in the same way after 1 h, with the treatment regimen illustrated in Fig. 5E. After LPS induction 6–12 h, the inflammatory response in mice is in a state of rapid activation, and the relevant inflammatory blood indicators will change significantly [50]. We evaluated the changes in the number of neutrophils in peripheral blood after treatment with different administration groups. The number and percentage of neutrophils remained basically consistent compared with the PBS group, which further supported the safety of neutrophil drugs (Fig. S15A and B). We collected serum from each treatment group for release of TNF-α, IL-1β, and IL-6 cytokines via Elisa assay after sepsis model establishment 12 h (Fig. 5F–H). Sepsis patients frequently have acute respiratory distress syndrome or severe lung damage, which is the leading cause of mortality [51]. Immunofluorescence labeling of TNF-α and IL-6 in mice lung tissues further confirmed that PBS group exhibited elevated TNF-α and IL-6 expression (the green or red areas represent positive expression) (Fig. S16). It was found that, the expression of these inflammatory factors was significantly suppressed in the MSe-NAD^+^/Nes group compared with the PBS group, which further confirms the protective effect of MSe-NAD^+^/Nes against inflammation in LPS-induced septic mice. These findings indicate that MSe-NAD^+^/Nes pre-treatment may improve the body's tolerance to inflammatory responses and reduce the degree of tissue damage during the state of inflammatory stress.

Serum biochemical markers were significantly elevated in the PBS group, confirming the successful establishment of the sepsis model. In sepsis mice, both MSe/Nes and NAD^+^/Nes demonstrated promising therapeutic potential, with particularly notable efficacy observed in the MSe-NAD^+^/Nes group. The inflammatory response in mice was further developed, and the relevant organs were damaged 12–24h after LPS induction. Serum was collected for ALT, AST, BUN, CREA and UA tests to assess the therapeutic effects on liver and kidney function in different mice. As shown in Fig. 5I–M, the liver and kidney function of the mice with sepsis significantly deteriorated. In spite of this, the liver and kidney function of mice with sepsis can still be effectively restored after MSe-NAD^+^/Nes treatment.

**Fig. 6:**
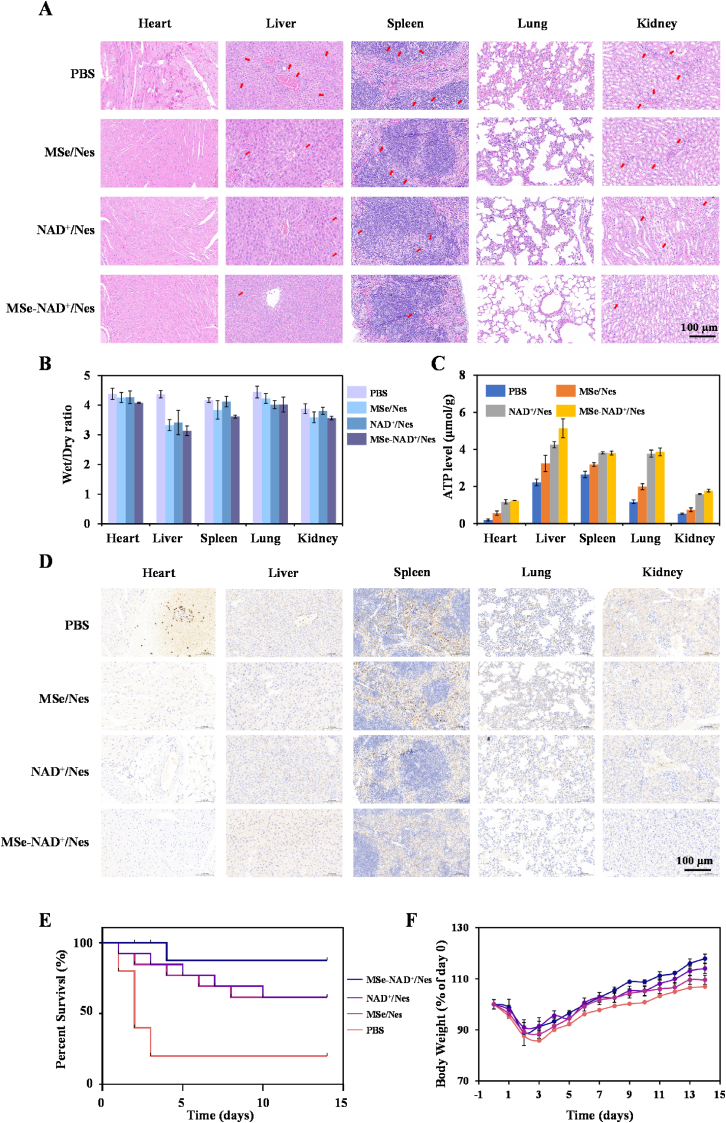
MSe-NAD^+^/Nes Alleviates Multiple-Organ Dysfunction and Inflammation of Sepsis Mice. The H&E staining of heart, liver, spleen, lung and kidney of PBS, MSe/Nes, NAD^+^/Nes and MSe-NAD^+^/Nes groups after treatment of sepsis mice (A); The wet/dry ratio of heart, Liver, Spleen, Lung and Kidney in sepsis mice (B); ATP levels in different organs of the mice treated with PBS, MSe/Nes, NAD^+^/Nes and MSe-NAD^+^/Nes groups (C); The immunohistochemical changes in NLRP3 in the heart, liver, spleen, lung, spleen, and kidney (D); Survival rate of sepsis mice within 14 days, data are mean ± SD, n = 10 (E); Body weight charts for 14 days of sepsis mice (F).

Given that multiple organ failure is a common clinical manifestation in severe sepsis, we evaluated the MSe-NAD^+^/Nes potential for repairing multi-organ damage through histopathological analysis. The organs of septic mice exhibited significant pathological changes, including inflammatory cell infiltration, thickening of alveolar walls, increased megakaryocyte counts, myocardial fiber disruption, hepatocyte necrosis, and renal tubular epithelial cell edema (Fig. 6A). Notably, after treatment with MSe-NAD^+^/Nes, the extent of damage to major organs in septic mice was significantly mitigated. In the sepsis mouse model, the wet/dry weight ratio of organs serves as a critical evaluation metric. This ratio reflects the relative water content within the organs and provides insight into the extent of edema and overall pathophysiological condition. By measuring the wet/dry weight ratio of major organs in different groups of mice, we found that the organs of sepsis model mice exhibited significant edema (Fig. 6B). Notably, the edema in the organs of mice treated with the MSe-NAD^+^/Nes was markedly reduced, indicating the protective effect of the MSe-NAD^+^/Nes on organ function. To further validate this hypothesis, ATP levels in major organs were measured following LPS treatment, as energy dysfunction is closely associated with sepsis-induced organ failure. Although LPS-induced inflammation impaired the energy supply in all major organs, MSe-NAD^+^/Nes significantly restored ATP levels, demonstrating its potent protective effect on organ function (Fig. 6C).

Then, we investigated the mechanisms underlying the therapeutic effects of MSe-NAD^+^/Nes on sepsis. NLRP3 immunohistochemical staining was performed on the major organs of each treatment group (Fig. 6D). In spleen tissue, NLRP3 expression was significantly higher in the PBS group (brown areas indicating positive expression). After treatment with MSe/Nes and NAD^+^/Nes, the brown-stained areas became progressively lighter and smaller, indicating reduced NLRP3 expression compared to the PBS group. Notably, the MSe-NAD^+^/Nes group showed the most significant reduction in NLRP3 expression. DHE, as a probe that can penetrate cell membranes for detecting ROS, can be oxidized into ethidium bromide with red color. The fluorescence intensity can be detected to indirectly reflect the level of ROS. Simultaneously, we conducted immunofluorescence staining to evaluate ROS levels in visceral organs (Fig. S17). The results showed a significant increase in ROS levels in the internal organs of septic mice. However, treatment with MSe/Nes and NAD^+^/Nes significantly reduced ROS levels, particularly in the MSe-NAD^+^/Nes group, indicating that MSe-NAD^+^/Nes treatment effectively alleviates organ oxidative stress. These findings demonstrate that after MSe-NAD^+^/Nes treatment, the inflammatory response, oxidative stress status and immune function in sepsis mice gradually returned to near-normal levels. This confirms that MSe-NAD^+^/Nes is a promising therapeutic option for sepsis.

Finally, we conducted a 14-day survival study and monitored weight changes throughout the experiment. As shown in Fig. 6E, the mortality rate in the PBS group reached 80 % within 3 days. The MSe/Nes and NAD^+^/Nes treatment group exhibited a slight improvement with 60 % survival at 14 days post-LPS induction. Notably, MSe-NAD^+^/Nes treatment significantly delayed mortality, achieving a survival rate of over 90 % even 4 days after LPS administration. This supports the protective effect of MSe-NAD^+^/Nes in acute sepsis models. Regular weight measurements revealed that untreated sepsis mice progressively lost weight until death. In contrast, the MSe-NAD^+^/Nes group showed the fastest weight recovery, indicating a gradual suppression of adverse reactions *in vivo* in Fig. 6F. As evidenced by the *in vivo* results, our drug delivery system effectively manages sepsis. MSe-NAD^+^/Nes not only exhibits excellent biosafety but also selectively targets inflammatory tissues, thereby efficiently controlling inflammation and oxidative stress while regulating energy metabolism to mitigate sepsis-induced organ damage. Whereas more intensive evaluations in different animals and after long-term treatment remain to be performed to fully demonstrate *in vivo* safety profiles of MSe-NAD^+^/Nes, these preliminary findings suggested that both MSe-NAD^+^/Nes are potentially safe at the examined doses. In summary, MSe-NAD^+^/Nes represents a promising therapeutic strategy for sepsis treatment.

## 4 Conclusion

In this study, we successfully developed MSe-NAD^+^/Nes, which can efficiently neutralize ROS and enhance mitochondrial homeostasis and cellular energy supply by effectively delivering NAD^+^ into cells. By leveraging the inflammatory targeting effects of neutrophils, the delivery of therapeutic agents via neutrophil carriers can enable targeted treatment of systemic inflammation and organ damage in sepsis. The delivery platform can effectively block the pathway between the inflammatory response and the reactive oxygen cycle process, thus effectively protecting normal cells from inflammatory damage. Meanwhile, it can inhibit NLRP3 inflammasome activation and attenuate the level of inflammatory factors, reduce the production of inflammatory factor storms, alleviate the condition of sepsis and effectively improve organ dysfunction. This research developed an effective and straightforward approach for treating sepsis while introducing innovative concepts for the advancement of nano-enzymatic therapeutic systems.

## CRediT authorship contribution statement

**Yingchun Zhao:** Writing – original draft, Data curation. **Ying Qu:** Formal analysis. **Changshun Huang:** Investigation. **Chengzhilin Li:** Methodology. **Wenyu Zhang:** Resources. **Xinyu Wang:** Software. **Wenlong Duan:** Supervision. **Qingbin He:** Validation. **Yachao Zhang:** Visualization. **Jianwei Jiao:** Resources. **Runxiao Zheng:** Writing – review & editing, Supervision.

## Declaration of competing interest

The authors declare that they have no known competing financial interests or personal relationships that could have appeared to influence the work reported in this paper.

## Data Availability

Data will be made available on request.
