# Supplementary material for: Neutrophils loaded NAD+ impede TLR4/NF-κB/NLRP3 pathway for sepsis treatment
Source: Mater Today Bio. 2025 Aug 4;34:102168. doi: 10.1016/j.mtbio.2025.102168 (PMC12345315; doi:10.1016/j.mtbio.2025.102168)
Supplement: Multimedia component 1 [file mmc1.docx]

**Supplementary information**

**Neutrophils loaded NAD^+^ impede TLR4/NF-κB/NLRP3 pathway for sepsis treatment**

*Yingchun Zhao^1^, Ying Qu^1^, Changshun Huang^1^, Chengzhilin Li^1^, Wenyu Zhang^1^, Xinyu Wang^1^, Wenlong Duan^1^, Qingbin He^1^, Yachao Zhang^1, 2*^, Jianwei Jiao^3^**^*^, Runxiao Zheng^1*^*

1. Shandong Provincial Hospital, Medical Science and Technology Innovation Center, Shandong First Medical University & Shandong Academy of Medical Sciences, Jinan, 250117, China.
2. Shandong Hongkui Medical Laboratory Co., Ltd. Jinan, 271100, China.
3. State Key Laboratory of Stem Cell and Reproductive Biology, Institute of Zoology, Chinese Academy of Sciences, Beijing 100101, China.

**Corresponding author:*

*Runxiao Zheng*

*Shandong Provincial Hospital, Medical Science and Technology Innovation Center, Shandong First Medical University & Shandong Academy of Medical Sciences, Jinan, Shandong 250117, China, zhengrunxiao@sdfmu.edu.cn*

*Jianwei Jiao*

*State Key Laboratory of Stem Cell and Reproductive Biology, Institute of Zoology, Chinese Academy of Sciences, Beijing 100101, China,* [*jwjiao@ioz.ac.cn*](mailto:jwjiao@ioz.ac.cn)

*Yachao Zhang*

*School of Life Sciences, Medical Science and Technology Innovation Center, Shandong First Medical University & Shandong Academy of Medical Sciences, Jinan, 250117, China, zhangyachao2007@163.com*

**Materials and methods**

**Chemotaxis of** **MSe-NAD^+^/Nes under inflammatory conditions**

To measure the migration capacity of neutrophils or neutrophil cyto-pharmaceuticals towards inflammation, a Transwell chamber with 3 mm pore size (Millipore, MA, USA) was used. The chambers were placed on a 24-well plate with each well containing 0.8 mL of completed RPMI 1640 medium with 100 nmol/L N-formyl-L-methionyl-L-leucyl-L-phenylalanine (fMLP). Then 1×10^6^ neutrophils, MSe-NAD^+^/Nes and MSe-NAD^+^ encapsulated by neutrophil cell membranes (mMSe-NAD^+^) were placed into the upper chamber, respectively, which was then incubated at 37 ℃ for 3 h. At the end of incubation, the lower chamber was imaged by an optical microscope (Nikon) and the number of migrated cells was analyzed by Image J.

Relative migrated cells (%) = The average number of cells in the lower chamber / The total number of feeding cells×100%

**Transfer of MSe-NAD^+^/Nes from neutrophils to macrophages**

To further determine the uptake of Nes, MSe-NAD^+^/Nes and mMSe-NAD+ by macrophages, RAW 264.7 cells were incubated with FITC-MSe-NAD^+^/Nes for 4 hours and stimulated with IL-1β (10 ng/mL). Neutrophils were labeled with APC-Ly6G, macrophage nuclei were stained with Hoechst 33342 and MSe-NAD^+^/Nes NPs were labeled with FITC. Confocal microscopy was employed to observe the phagocytic activity of macrophages. In addition, we inoculated RAW 264.7 cells in the lower layer of transwell chambers. At regular intervals, RAW 264.7 cells in the lower chamber of the transwell were harvested. Subsequently, the cells in the lower chamber were gently

rinsed 2-3 times with PBS to eliminate the un-internalized fluorescent drugs. After that, an appropriate volume of cell lysis buffer was added to the washed cells in the lower chamber. The lysate was then centrifuged, and the supernatant was collected for subsequent detection of fluorescence intensity.

**Immunofluorescence staining**

RAW264.7 cells were treatment with different group treatment, fixed by 4% paraformaldehyde for 15 min at 4°C, followed by permeabilization with 0.1% Triton X-100. After blocking, cells were incubated with the anti-TNF-α, anti-IL-1β rabbit polyclonal antibody overnight at 4 °C, respectively. Then, above treated macrophages were washed with PBS for 3 times. Meanwhile, cells were stained with Coralite Plus 488-labeled goat anti-rabbit IgG secondary antibody for another 1 h. In addition, DAPI was used to label cell nuclear, and image acquisition under a fluorescence microscope (OLYMPUS TH4-200, ZEISS). The lung tissue sections from mice were deparaffinized, and then the antigen was detected using an EDTA antigen retrieval buffer (pH 8.0). Then they were inhibited for 60 min with 5% BSA. After that, sections were treated with primary antibodies against TNF-α (1:100) and IL-6 (1:100) overnight at 4℃. Then, fluorescent secondary antibodies labeled with FITC (1:100, Servicebio, China) was added, pictures were taken using a slide scanner.

**Figure S1.** The pore size distributions of MSe NPs derived from desorption isotherm measurements and BJH methods.

**Figure S2**. Stability study of the MSe-NAD^+^ NPs. (A) MSe-NAD^+^ NPs is dispersed in deionized water or culture medium, and their sizes were monitored by DLS at different time points; (B) Polydispersity coefficient of MSe-NAD^+^ NPs in deionized water or culture medium for 1 week at room temperature. Data are expressed as mean ± S.D. (n=3). Statistical significance was calculated via one-way ANOVA with Tukey post hoc test.

**Figure S3.** (A) Flow cytometric analysis of the purity of isolated neutrophils. The isolated neutrophils were double-stained with FITC anti-mouse CD11b and APC anti-mouse Ly6G antibodies; (B) Morphological images of isolated neutrophils stained with Giemsa Wright stain. Scale bar: 5 mm.

**Figure S4.** (A) Percentage of migrated neutrophils in the lower chamber. Fresh neutrophils (Nes) were used as control; (B) Confocal analysis of the uptake ability of macrophages for neutrophils; The nuclei of RAW264.7 were stained with Hoechst 33342 (Blue), MSe-NAD^+^ NPs were stained with FITC (Green), and neutrophils was labeled with APC-Ly6G (Red); (C) The fluorescence intensity of RAW264.7 cells in the inferior cavity stimulated by IL-1β after incubation with MSe-NAD^+^/Nes.

**Figure S5**. (A) The GPx-like activity of MSe NPs and GPx at pH 7.4 and 5.0; (B) The GPx-like activity of MSe NPs and GPx at 25°C and 60°C; (C) H_2_O_2_ elimination efficiency of MSe NPs, and natural GPx pretreated at pH 7.4 and 5.0; (D) H_2_O_2_ elimination efficiency of MSe NPs, and natural GPx pretreated at 25°C and 60 °C.

**Figure S6**. (A) Elimination of ·OH in MSNs, NAD^+^, MSe NPs and MSe-NAD^+^ NPs; (B) Elimination of ·O_2_^-^ in MSNs, NAD^+^, MSe NPs and MSe-NAD^+^; (C) The dependence of different concentration MSe NPs on the ·OH elimination; (D) The elimination of ·OH efficiency of MSe NPs; (E) The dependence of different concentration of MSe NPs on the ·O_2_^-^ elimination; (F) The elimination of ·O_2_^-^ efficiency of MSe NPs.

**Figure S7**. (A) CVs of the GCE electrode modified with as-prepared MSNs, NAD^+^, MSe NPs and MSe-NAD^+^ NPs presence of 1.00 mM H_2_O_2_, the unmodified GC electrode was used as a control; (B) Current density of different samples at the potential of 1.06 V obtained from A.

**Figure S8.** The NAD^+^/NADH ratio of RAW264.7 cells in control, PBS, NAD^+^/Nes, MSe/Nes and MSe-NAD^+^/Nes in the LPS-induced energy consumption model. The group without LPS stimulation was taken as the control group. RAW264.7 cells were treated with LPS (100 ng/mL) and then treated with PBS, MSe/Nes, NAD^+^/Nes and MSe/NAD^+^/Nes respectively (equivalent to 25µg/mL MSe NPs or 6.5 µg/mL NAD^+^). Values are expressed as mean ± S.D. ^**^ *p* < 0.01, ^*^ *p* < 0.05 vs. control group, ^##^ *p* < 0.01, ^#^ *p* < 0.05 vs. PBS group.

**Figure S9**. Determination of fluorescein-annexin V and PI staining after treatment with control, PBS, NAD^+^/Nes, MSe/Nes and MSe-NAD^+^/Nes, scare bar = 100 μm. The group without LPS stimulation was taken as the control group. RAW264.7 cells were treated with LPS (100 ng/mL) and then treated with PBS, MSe/Nes, NAD^+^/Nes and MSe/NAD^+^/Nes respectively (equivalent to 25µg/mL MSe NPs or 6.5 µg/mL NAD^+^).

**Figure S10**. JC-1 aggregates/monomers Fluorescence ratio. The group without LPS stimulation was taken as the control group. RAW264.7 cells were treated with LPS (100 ng/mL) and then treated with PBS, MSe/Nes, NAD^+^/Nes and MSe/NAD^+^/Nes respectively (equivalent to 25µg/mL MSe NPs or 6.5 µg/mL NAD^+^). Values are expressed as mean ± S.D. ^**^ *p* < 0.01, ^*^ *p* < 0.05 vs. control group, ^##^ *p* < 0.01, ^#^ *p* < 0.05 vs. PBS group.

**Figure S11.** Determination of fluorescence images results of intracellular ROS after treatment with control, PBS, NAD^+^/Nes, MSe/Nes and MSe-NAD^+^/Nes, scare bar = 200 μm. The group without LPS stimulation was taken as the control group. RAW264.7 cells were treated with LPS (100 ng/mL) and then treated with PBS, MSe/Nes, NAD^+^/Nes and MSe/NAD^+^/Nes respectively (equivalent to 25µg/mL MSe NPs or 6.5 µg/mL NAD^+^).

**Figure S12.** The relative fluorescence ratio of different nanozymes of Mitosox Red. The group without LPS stimulation was taken as the control group. RAW264.7 cells were treated with LPS (100 ng/mL) and then treated with PBS, MSe/Nes, NAD^+^/Nes and MSe/NAD^+^/Nes respectively (equivalent to 25µg/mL MSe NPs or 6.5 µg/mL NAD^+^). Values are expressed as mean ± S.D. ^**^ *p* < 0.01, ^*^ *p* < 0.05 vs. control group, ^##^ *p* < 0.01, ^#^ *p* < 0.05 vs. PBS group.

**Figure S13**. Uncroppoed western blots of TLR4, Phospho-NF-κB p65, NF-κB p65, NLRP3 and β-actin of RAW264.7 cells treated with MSe/Nes, NAD^+^/Nes, and MSe/NAD^+^/Nes (equivalent to 25 µg/mL MSe NPs or 6.5 µg/mL NAD^+^), together with LPS (100 ng/mL). Without LPS challenge were used as control groups.

**Figure S14.** (A) Immunofluorescence staining of TNF-α and IL-1β in different treatment groups; (B) The relative fluorescence intensity of TNF-α in different treatment groups; (C) The relative fluorescence intensity of IL-1β in different treatment groups. The group without LPS stimulation was taken as the control group. RAW264.7 cells were treated with LPS (100 ng/mL) and then treated with PBS, MSe/Nes, NAD^+^/Nes and MSe/NAD^+^/Nes respectively (equivalent to 25µg/mL MSe NPs or 6.5 µg/mL NAD^+^). Values are expressed as mean ± S.D. ^**^ *p* < 0.01, ^*^ *p* < 0.05 vs. control group, ^##^ *p* < 0.01, ^#^ *p* < 0.05 vs. PBS group.

**Figure S15.** Changes in the levels of blood cells of mice after LPS Induction. (A) Whole blood analysis of neutrophils and (B) percentage of neutrophils. WBC, white blood cells; Data are mean ± S.D.

**Figure S16.** Lung tisues immunoluorecence of inammatory factors TNF-α and IL-6 24 h after LPS. scale bar = 100 μm.

**Figure S17**. Dihydroethidium (DHE) staining of Heart, Liver, Spleen, Lung and kidney tissue in different groups (red fluorescence: DHE; blue fluorescence: cell nucleus), scare bar = 100 μm.
